# Brain Age Prediction With Morphological Features Using Deep Neural Networks: Results From Predictive Analytic Competition 2019

**DOI:** 10.3389/fpsyt.2020.619629

**Published:** 2021-01-20

**Authors:** Angela Lombardi, Alfonso Monaco, Giacinto Donvito, Nicola Amoroso, Roberto Bellotti, Sabina Tangaro

**Affiliations:** ^1^Istituto Nazionale di Fisica Nucleare, Bari, Italy; ^2^Dipartimento Interateneo di Fisica, Università degli Studi di Bari Aldo Moro, Bari, Italy; ^3^Dipartimento di Farmacia - Scienze del Farmaco, Universitá degli Studi di Bari Aldo Moro, Bari, Italy; ^4^Dipartimento di Scienze del Suolo, della Pianta e degli Alimenti, Università degli Studi di Bari Aldo Moro, Bari, Italy

**Keywords:** brain aging, deep neural networks, machine learning, MRI, FreeSurfer, morphological features, aging biomarker

## Abstract

Morphological changes in the brain over the lifespan have been successfully described by using structural magnetic resonance imaging (MRI) in conjunction with machine learning (ML) algorithms. International challenges and scientific initiatives to share open access imaging datasets also contributed significantly to the advance in brain structure characterization and brain age prediction methods. In this work, we present the results of the predictive model based on deep neural networks (DNN) proposed during the Predictive Analytic Competition 2019 for brain age prediction of 2638 healthy individuals. We used FreeSurfer software to extract some morphological descriptors from the raw MRI scans of the subjects collected from 17 sites. We compared the proposed DNN architecture with other ML algorithms commonly used in the literature (RF, SVR, Lasso). Our results highlight that the DNN models achieved the best performance with *MAE* = 4.6 on the hold-out test, outperforming the other ML strategies. We also propose a complete ML framework to perform a robust statistical evaluation of feature importance for the clinical interpretability of the results.

## 1. Introduction

The last few decades have seen significant advances in neuroimaging methodologies and machine learning (ML) techniques focused on identifying structural and functional features of the brain associated with the age. Age prediction is typically performed using a multivariate set of features derived from one or multiple imaging modalities. A dataset is then specified by including the characteristics of different subjects and their chronological ages. The dataset is employed to train one or more supervised machine learning algorithms which attempt to predict a given subject's brain age by using the brain imaging features while minimizing the difference from the true age and preventing overfitting. Different metrics are commonly used to assess the delta between the predicted age and the actual age of the participants (i.e., the brain age gap), such as Mean Absolute Error (MAE) (1).

A great variety of ML approaches including deep learning techniques have been proposed to predict age from brain magnetic resonance imaging (MRI) scans (2, 3). Typically, a number of selected features are extracted from images such as morphological descriptors, complex network-based models or radiomic features (4–7) or raw high-dimensional data are exploited to feed more complex models such as convolutional neural networks (8–10). One of the most promising uses of the brain age prediction is its relevance and use as a biomarker to assess the risk of an individual to develop cognitive decline and propensity to neurodegenerative diseases (11–13). The idea underlying this approach is that the age gap could be a reliable clinical marker as it has been related to abnormal age changes in different pathologies such as schizophrenia (14), Alzheimer's disease (15), traumatic brain injury (16).

In order to ensure both generalization and reliability, the ML algorithms should return accurate responses on unseen datasets. However, choosing a model suitable for heterogeneous dataset requires high computational complexity and extensive evaluation of parameter combinations. International competitions facilitate the comparison of different techniques on large datasets favoring a deeper comparison of algorithms and classification strategies with transparent procedures and data sharing policies (17–19).

In this work, we present the results of the predictive model based on deep neural networks (DNN) proposed during the Predictive Analytic Challenge 2019 for brain age prediction of healthy individuals by using some morphological descriptors extracted from their raw MRI scans. Recently we have used a set of morphological features to describe the trajectories of neurodevelopment on a cohort of ABIDE database (20), proving the efficiency of this representation for brain age prediction in a limited age range (21). In this paper we propose a different architecture and a machine learning framework for a more in-depth comparison with other machine learning techniques commonly used in the literature. Another fundamental objective of the work is to provide a robust statistical evaluation of feature importance for the explanation of the results obtained with the DNN models in order to facilitate their inclusion in clinical contexts.

## 2. Materials

### 2.1. Subjects

In this study, we included 2638 T1-weighted MRI brain images collected from 17 sites and provided by the organizers of Predictive Analytic Competition (PAC) 2019^1^. This competition consisted of two sub challenges: (i) to achieve the lowest mean absolute error for brain age prediction; (ii) to achieve the lowest MAE while keep the Spearman correlation between the brain-age delta and the chronological age under 0.1. We processed the T1 raw images with FreeSurfer software on ReCaS Datacenter as described in section 2.2. After the preprocessing step, 478 subjects were excluded from the next steps of the analysis either because of pipeline failure or because they were marked as outliers during the quality assessment step of the features extracted from the pipeline. The demographic characteristics of the remaining 2,170 subjects are listed in Table 1 for each of the 17 sites.

**Table 1:** Demographic information of the subjects per site.

**Site**	**Samples**	**Age (years)**	**Gender (M/F)**
0	304	34.1 ± 12.6	120/184
1	129	26.9 ± 9.3	53/76
2	492	35.4 ± 12.3	211/281
3	140	25.5 ± 6.6	122/18
4	131	21.3 ± 2.0	52/79
5	35	31.5 ± 7.7	15/20
6	9	62.4 ± 7.1	7/2
7	23	43.1 ± 11.4	8/15
8	156	24.7 ± 5.2	68/88
9	415	49.2 ± 16.7	180/235
10	73	32.9 ± 11.2	51/22
11	18	69.9 ± 7.9	9/9
12	29	29.2 ± 7.9	15/14
13	115	40.6 ± 17.2	70/45
14	56	41.7 ± 19.2	17/39
15	17	23.2 ± 1.2	3/14
16	28	22.9 ± 2.8	8/20

### 2.2. Morphological Features

As in our previous work (21), we exploited ReCaS datacenter^2^ to create a custom pipeline for preprocessing and analysis of T1 raw images (22). The ReCaS-Bari computing farm has been built by the ReCaS project^3^, funded by the Italian Research Ministry of Education, University and Research to the University of Bari and INFN (National Institute for Nuclear Physics) and offers a complete scientific high-throughput and high- performance computing environment to deal with common problems of large-scale neuroimaging processing.We integrated the software tool FreeSurfer^4^ into a pipeline to extract the morphometric properties of both cortical and sub-cortical brain structures. In particular, the morphological features were extracted by using the FreeSurfer v.6.0.0 recon-all pipeline (23–25). The recon-all workflow allows for the fully automated cortical and sub-cortical segmentation and reconstruction by using several steps such as motion correction, non-uniform intensity normalization, transform in Talairach space, intensity normalization, skull stripping, cortical and sub-cortical parcellation. More details about all the steps included into the pipeline can be found at the web page of the pipeline^5^. The Desikan-Killiany atlas (26) was adopted for the cortical segmentation of each MRI scan into 68 anatomical regions of interest and the Aseg Atlas (25) for the sub-cortical segmentation into 40 regions of interest. The recon-all pipeline returns a list of metrics that statistically describe both the intensity- related and morphometric properties of the segmented regions. In particular, here we considered the following statistical features:

- Volume of 40 sub-cortical brain structures (40 features included in aseg.stats file);
- Volume of white matter parcellation of brain cortex (68 features included in wmparc.stats file);
- Volume, surface area, mean curvature, mean thickness for the 34 cortical brain regions of each hemisphere (272 features included in aparc.stats file);
- Global brain metrics including surface and volume statistics of each hemisphere; total cerebellar gray and white matter volume, brainstem volume, corpus callosum volume, white matter hypointensities (9 features included in wmparc.stats, aparc.stats and aseg.stats files).

ReCaS scientific environmental offers some facilities to perform quality check and output verification of the implemented pipelines by integrating information extracted in log files and crash files. Specifically, the quality assessment of the resulting features was performed by excluding extreme outliers through the MAD criterion (27) and subjects on which some pipeline steps have failed. At the end of this stage, we constructed a matrix of features *N* × *P* with *N* = 2, 170, and *P* = 389, where each row represents a single subject described with *P* morphological features.

## 3. Methods

### 3.1. Machine Learning Framework

**Figure 1:**
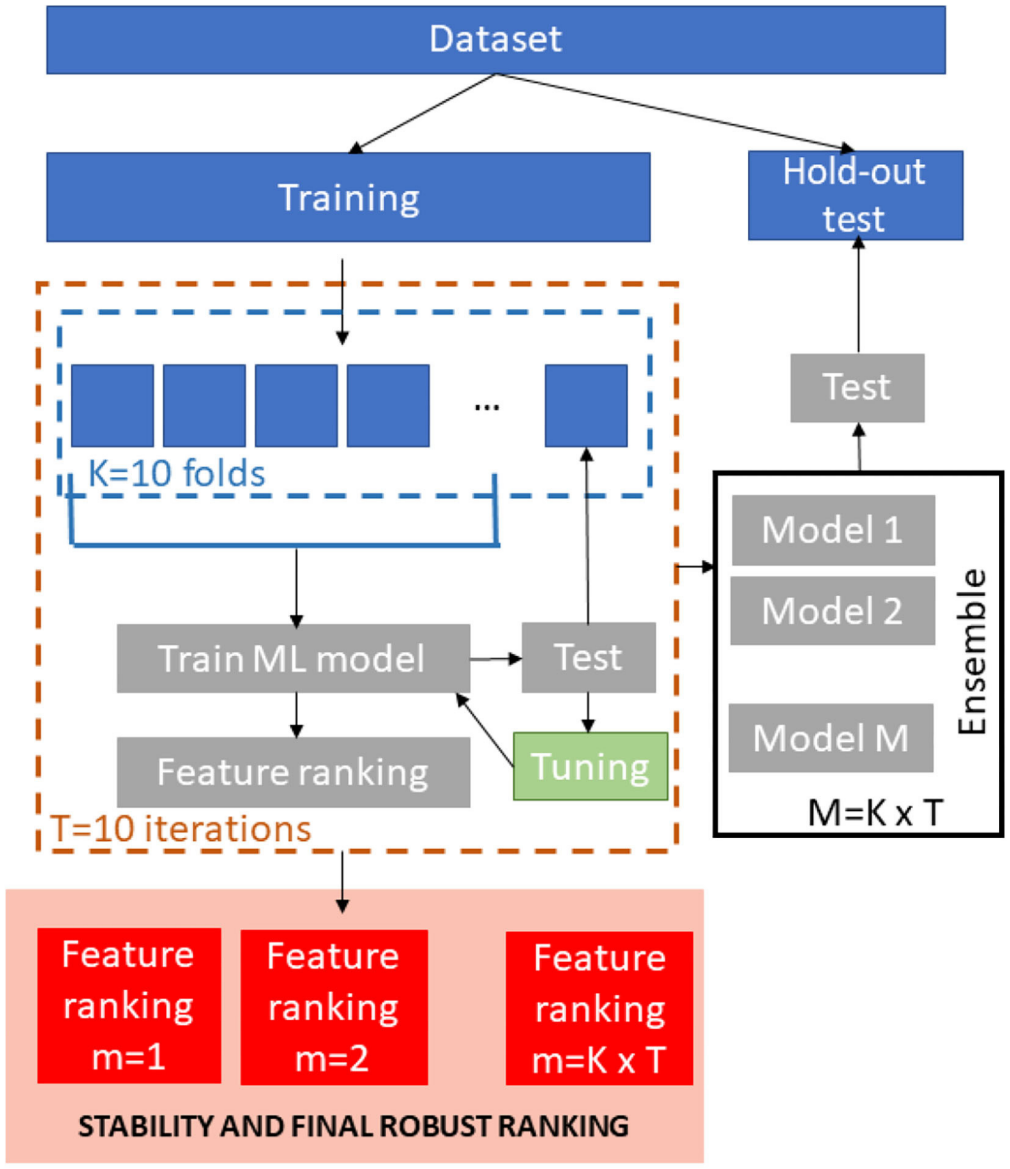
Schematic overview of the ML framework.

A schematic overview of the ML framework is shown in Figure 1. We stratified the age values in order to obtain a representative test sample so the database was divided into training set (1,500 subjects) and hold-out independent test (760 subjects).

For the training phase, *T* = 10 re-sampling of a *K* = 10-fold cross-validation were executed producing 100 bootstraps of the training dataset. In each iteration, nine-folds of the dataset were input to four different regression models (Support vector Regression, Random Forest, Lasso and Deep Neural Networks). We performed the same min-max normalization procedure on the training set within each round and applied the parameters to normalize the left fold. For the Random Forests and Support Vector Regression models, we trained stepwise models for ranked subsets of increasing size obtained by using embedded and recursive feature elimination (RFE) algorithms, respectively. The performance of the each model was evaluated on the left test fold. The main goal of this stepwise analysis was to detect the specific subset of features that minimizes the averaged prediction error (28). As a result, this step returns the optimum number of non-redundant features *k*_*opt*_ to retain in order to achieve the best performance and the best performing model for this set of features. For Lasso and DNN models, we trained a single model within each cross-validation round that was tested on the left fold in order to tune the model parameters since these methods perform an embedded selection of the best features.

For each regression algorithm, we applied an ensemble strategy by testing each of the final 100 models on the hold-out independent test and by averaging the resulting predictions to obtain the final age of each subject.

The best performing algorithm for age prediction was identified by comparing the performance of all the models. We also compared the sets of ranked features across models by using a stability index for the clinical interpretation of the results. Each step of the framework is described in the following sections more in details.

### 3.2. ML Regression algorithms

The four different regression models support vector regression (SVR), random forest (RF), Lasso and deep neural networks (DNN) were evaluated to predict brain ages of *N* subjects *Y* ∈ ℝ^*N*^ based on the matrix of predicting variables *X* ∈ ℝ^*N*×*P*^. To evaluate the regression performance, two different metrics were employed:

- Mean Absolute Error (MAE):

(1) $$\begin{array}{r} MAE=\frac{1}{N}\overset{N}{\underset{i=1}{\sum }}|y_{i}-\hat{y_{i}}| \end{array}$$

- Pearson correlation coefficient (*R*):

(2) $$\begin{array}{r} R=\frac{\sum_{i=1}^{N}\left(y_{i}-ȳ\right)\left(\hat{y_{i}}-\bar{ŷ}\right)}{\sqrt{\sum_{i=1}^{N}{\left(y_{i}-ȳ\right)}^{2}}\sqrt{\sum_{i=1}^{N}{\left(\hat{y_{i}}-\bar{ŷ}\right)}^{2}}} \end{array}$$

with N being the sample size, *y*_*i*_ the chronological age, $\hat{y_{i}}$ the predicted brain age and *ȳ* and $\bar{ŷ}$ denote their sample means.

#### 3.2.1. Support Vector Regression

Support vector regression (SVR) is a machine learning algorithm that aim to determine a cost function *f* (*x*) with deviations ϵ_*n*_ < ϵ from each target point *y*_*n*_ and each training point *x*_*n*_ (29).

It represents a kernel-base method that can also be viewed as a linear regression into a higher dimensional space in which the data are mapped through a non-linear kernel function (30). In our analysis we applied the SVR implementation of the “Caret” R package^6^ with linear kernel and the default parameters (ϵ = 0.1).

For feature ranking we applied the Support Vector Machine-Recursive Feature Elimination (SVM-RFE) algorithm as it is able to perform both feature selection and regression task. Indeed, this algorithm requires that firstly the regression model is trained, then the ranking of all features is determined and lastly the features with the smallest ranking criterion are excluded from the initial list. This process is reiterated until all the features have been removed from the list (31).

#### 3.2.2. Random Forest

Random forest (RF) algorithm is an ensemble of tree-based base learners. The target outcome is independently predicted by each tree, while the final predictions are based on the average of individual tree predictions (32). They are constructed by introducing randomness as a subset of observations is randomly selected for each tree and a random set of *mtry* candidate predictors is selected to create a split within each tree. The node input samples are divided into two sets according to a purity metric and a decision threshold and each tree is grown until nodes have split their inputs into subsets with a single label. The samples not used for a specific tree are comprised in the out of the bag (OOB) set for that tree. The samples of the OOB set are used to assess the accuracy of RF as:

(3) $$\begin{array}{r} OOB-MSE=\frac{1}{n}\overset{n}{\underset{i=1}{\sum }}{\left(y_{i}-\bar{\hat{y_{iO}}}\right)}^{2} \end{array}$$

where $\bar{\hat{y_{iO}}}$ denotes the average prediction for the *ith* observation from all trees for which this observation has been OOB.

We computed the RF feature importance by applying the permutation-based MSE reduction criterion (33). The importance of each feature in each tree is assessed by permuting the OOB data of the feature for the tree and by computing the difference between the permuted and the actual OOB-MSE. The final MSE reduction for each features is obtained by averaging these differences over all the trees of the forest. The main rationale of this approach is that if a feature does not affect the performance, the difference between the accuracy computed with the actual values of the feature and that computed by using its permuted values is expected not to be significant.

We used the “RandomForest” R Package^7^ with the default parameter *mtry* = *P*/3 and *ntree* = 500.

#### 3.2.3. Lasso

Lasso (Least Absolute Shrinkage and Selection Operator) is a regression method introduced by (34) to solve issues related to overfitting and multicollinearity in ordinary least square regression (OLS). In this method a penalty term is introduced to control the complexity of the model which is optimized for sparseness. Hence, the coefficients of the least significant features are shrinked to zero. This algorithm is also applied for feature selection as the subset of the features with non-zero weights can be extracted as an outcome of the model.

Lasso minimizes the residual sum of squares (RSS) to find the weights of the features:

(4) $$\begin{array}{r} RSS=\frac{1}{2}||Y-\beta X{||}_{2}^{2}-\lambda ||\beta {||}_{1} \end{array}$$

We used the Lasso implementation in “Caret” R package. The inner round of each fold of the cross validation has been used to find the best value of λ by searching in the range [10^−4^, 10^4^] with step 0.1.

In addition, since the absolute values of the Lasso coefficients could be used to find the number of useful features, we exploited both the frequency of occurrence of non-zero weights and their averaged absolute value across the validation rounds to identify the features most representative of the population, regardless the specific training fold.

#### 3.2.4. Deep Neural Networks

In this work we adopted a feed-forward deep neural networks. This class of networks comprise multiple layers of computational neurons, interconnected in a feed-forward way. Each neuron in one layer form connections with the neurons of the subsequent layer (35). This DNN architecture was implemented with the “h2o” R package^8^. We performed a grid search optimization provided by the “h2o” package on the inner round of each fold of the cross validation in order to reach a stable configuration by setting number of layers, neurons per layer and activation function. We obtained the final configuration with four hidden layers respectively including 256, 128, 56, and 24 neurons with linear rectifier (i.e., ReLU) as activation function.

In order to avoid overfitting, we adopted the default values provided by “h2o” R package for all the remaining parameters. In details, as described on the reference manual, h2o implements an adaptive learning rate for the stochastic gradient descent optimization. This methods depends on two parameters that control the balance of global and local research efficiencies: ρ is the similarity to prior weight updates and ϵ prevents the optimization to get stuck in local optima. Defaults values used in this work are ρ = 0.99 and ϵ = 10^−8^. In addition, the weights were randomly initialized within each cross-validation round to increase the network robustness.

The Gedeon method (36) was employed to obtain a ranked list of features. This algorithm considers the weight matrices connecting the inputs with the first two hidden layers to compute the relative importance of each variable.

### 3.3. Feature Importance

At the end of all cross-validation rounds, we obtained a matrix of ranked features *N* × *P* for each regression algorithm. This matrix has been analyzed to compute a features ranking representative of the whole population and independent from the specific cross-validation round. A consensus ranking algorithm was applied to select the most stable features across all the 100 cross- validation rounds. The main goal of a consensus ranking algorithm is to assess the stability of a ranked list of features with regard to minor alterations in the training sets drawn from the sample distribution (37). In particular, the robust rank aggregation (RRA) algorithm has proved to be one of the most effective to assess the final aggregated ranked list of multiple base rankers (38). Indeed, this approach computes the list of statistically significant items in the final ranking by comparing the position of each item in all the ranked lists to a null model of random permutations of the items. Here we extracted the final ranked list of features for each regression algorithm by applying the RRA method and then we evaluated the overlap between each couple of ranked list resulting from the different ML algorithms.

For Lasso algorithm, we also verified the correspondence among the final ranked set and the most important features obtained with the embedded frequency- and weights-based criterion.

The percentage of overlap between two set of features was computed through the Jaccard index as:

(5) $$\begin{array}{r} J\left(A,B\right)=\frac{\left|A\cap B\right|}{\left|A\cup B\right|} \end{array}$$

where *A* and *B* are two sets of ranked features. This index expresses the consensus between the two sets of features and is closely linked to the stability of the selected features with respect to the machine learning algorithms (39). Since 0 ≤ *J* ≤ 1, a higher percentage of overlap between the two sets means that the selected features are more invariant with respect to the ML algorithm.

## 4. Results

### 4.1. Cross-Validation Performance

**Figure 2:**
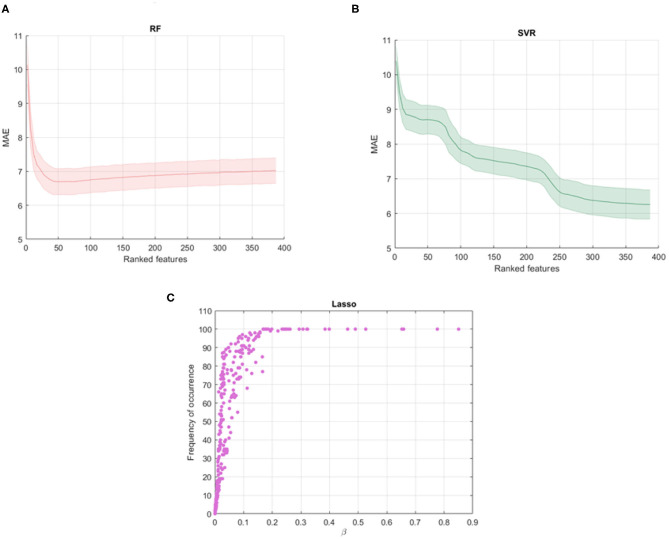
Shadow performance curves with the average MAE achieved by all the stepwise models in cross-validation with the standard errors for **(A)** RF models and **(B)** SVR models; **(C)** averaged weights vs. frequency of occurrence of the features across all the validation rounds resulting from Lasso algorithm.

Figures 2A,B show the average MAE values and standard deviations for different subset of the ranked features obtained with the RF and SVR algorithms. It is interesting to note that RF shows a decay in performance after a minimum peak reached for *k*_*opt*_ = 40 and therefore the other features are poorly informative and redundant. On the other hand, SVR shows the best performance for all the ranked features so we selected the 100 cross-validated RF models for *k*_*opt*_ = 40 and the 100 cross-validated SVR models for *k*_*opt*_ = 389. Figure 2C shows the averaged β weights and the frequency of occurrence of the features across the validation rounds for Lasso algorithm. Among the total set, 32 features were identified with averaged weights above the 90th upper percentile of the distribution of the averaged beta values across the rounds. These features also present a 100% frequency of occurrence meaning that they are selected in all the cross-validation rounds. The Gedeon algorithm returns the relative importance of each feature using a posterior evaluation of the net weights, so a specific subset from the total set of the features was not identified, setting *k*_*opt*_ = 389.

**Figure 3:**
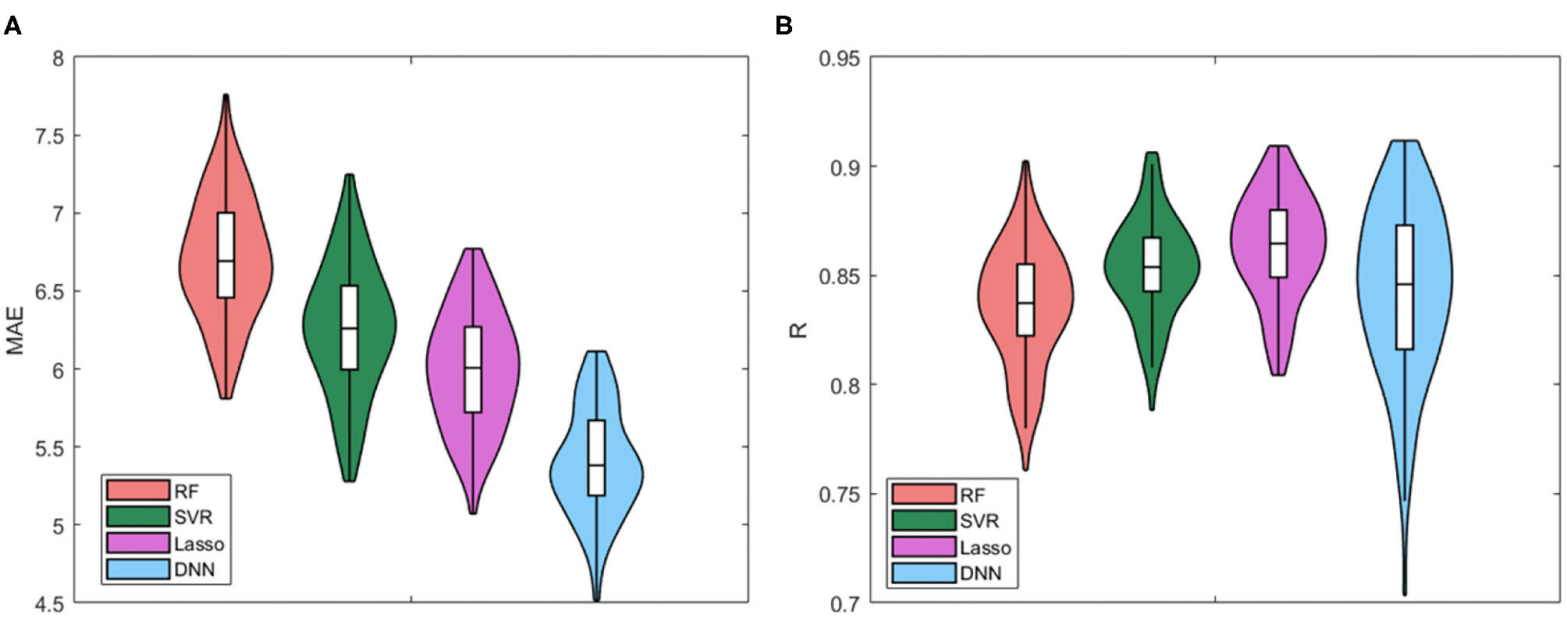
Violin plots of the distributions of **(A)** MAE values and **(B)** R values for the four models.

We compared all the cross-validated models for the four regression algorithms. The Violin plots of the distributions of MAE values and R values for the four models are presented in Figure 3. Table 2 also summarizes the mean and standard deviation values of the two performance metrics. The best performance is achieved by using the DNN algorithm, which MAE values resulted significantly different from the other distributions (*p* < 0.001 for Bonferroni *post-hoc* test). There are no substantial differences between the distributions of Pearson's values among the algorithms, while RF resulted the worst regression method for both performance metrics.

**Table 2:** Mean MAE ± SD resulting from age prediction in cross-validation rounds for the four regression models RF, SVR, Lasso, and DNN.

**Model**	**MAE**	**R**
RF	6.71 ± 0.39	0.83 ± 0.02
SVR	6.25 ± 0.41	0.85 ± 0.02
LASSO	5.99 ± 0.36	0.86 ± 0.02
DNN	5.39 ± 0.34	0.84 ± 0.03

**Figure 4:**
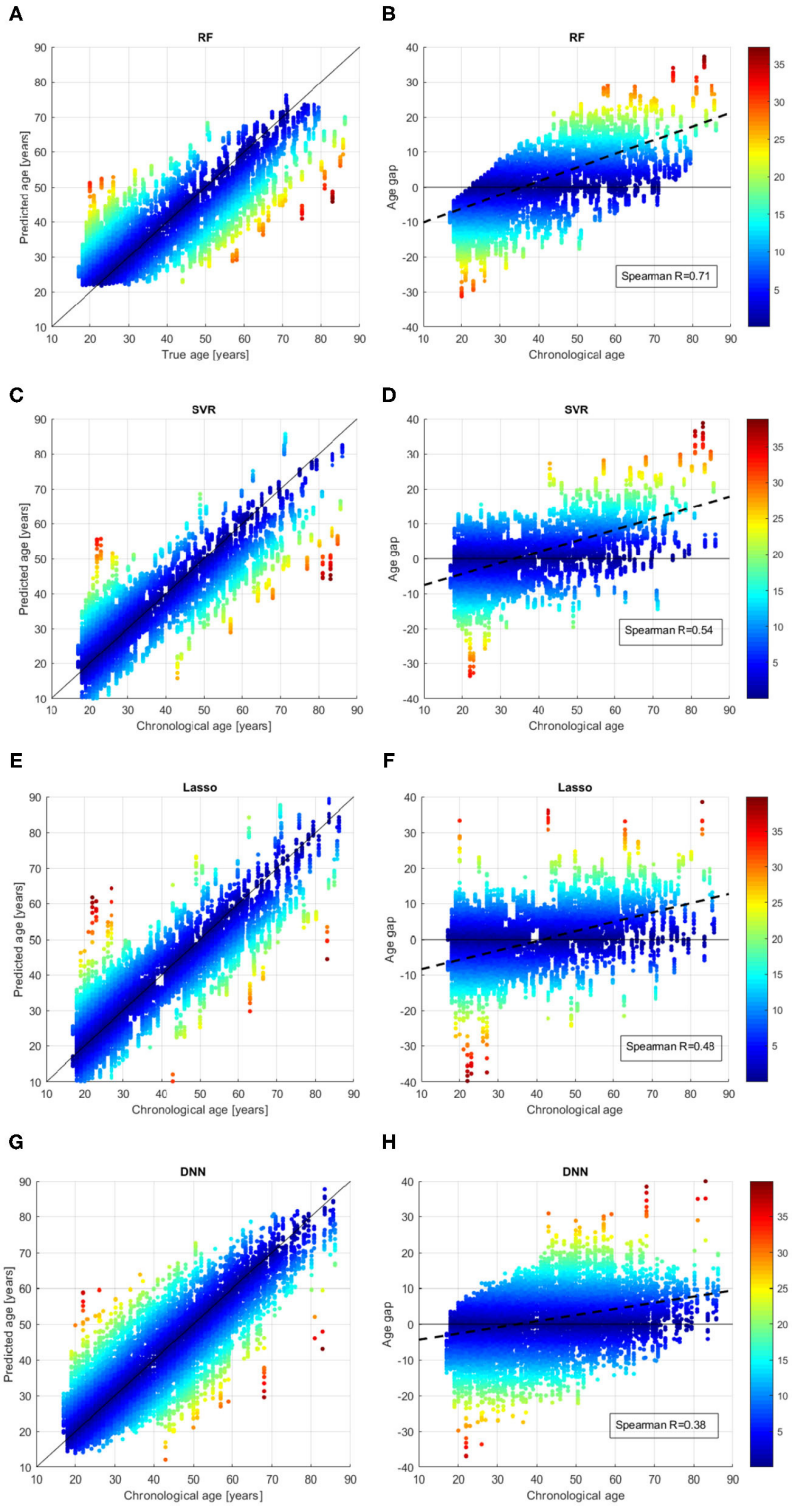
Results of brain age prediction for the training set in cross-validation rounds for **(A)** the RF model, **(C)** the SVR model, **(E)** the Lasso model, **(G)** the DNN model; results of age gap (Δ) for the training set in cross-validation rounds for **(B)** the RF model, **(D)** the SVR model, **(F)** the Lasso model, **(H)** the DNN model.

We better analyzed the behavior of the ML algorithms on the training set, by inspecting the comparison between the chronological age and the predicted age for each sample across all the validation rounds as shown in Figure 4. We also evaluated the age bias of the models, by considering the age gap Δ = *chrnologicalage*−*predictedage* vs. the chronological age of the subjects in the training set (see Figures 4B,D,F,H). The color of each point represent the absolute value of the age gap resulting from each validation round. All models exhibit samples with high age error in the first range (*age* < 25 years) or in the last range (*age* > 80 years), however the DNN models show the lowest age bias reporting Spearman coefficient *R* = 0.38.

### 4.2. Hold-Out Test

**Figure 5:**
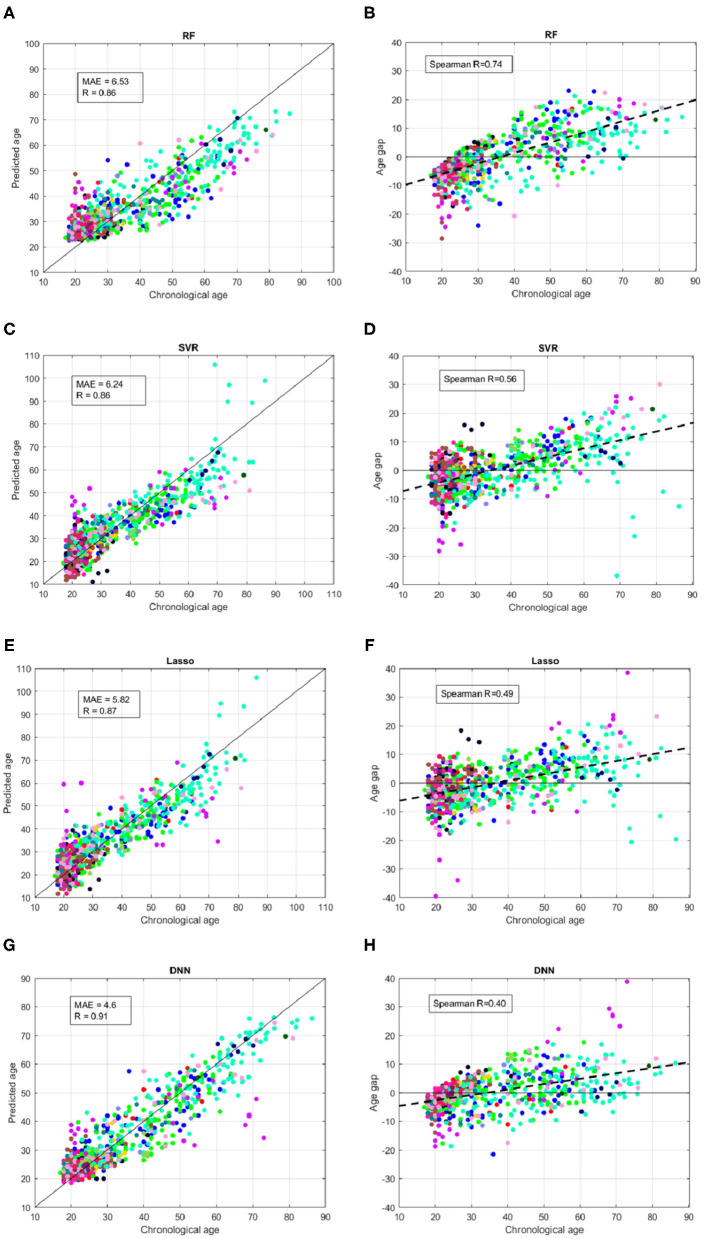
Results of brain age prediction for the independent test set for **(A)** the RF model, **(C)** the SVR model, **(E)** the Lasso model, **(G)** the DNN model; results of age gap (Δ) for the independent test set for **(B)** the RF model, **(D)** the SVR model, **(F)** the Lasso model, **(H)** the DNN model. Each color indicates a specific site.

Figure 5 summarizes the performance of the models on the independent hold-out test. We used different colors for the 17 sites of the subjects. Similarly to the cross-validation, the DNN resulted the best models for both age prediction and age bias, reporting *MAE* = 4.6, Pearson correlation *R* = 0.91 between the chronological age and predicted age and Spearman coefficient *R* = 0.4.

**Figure 6:**
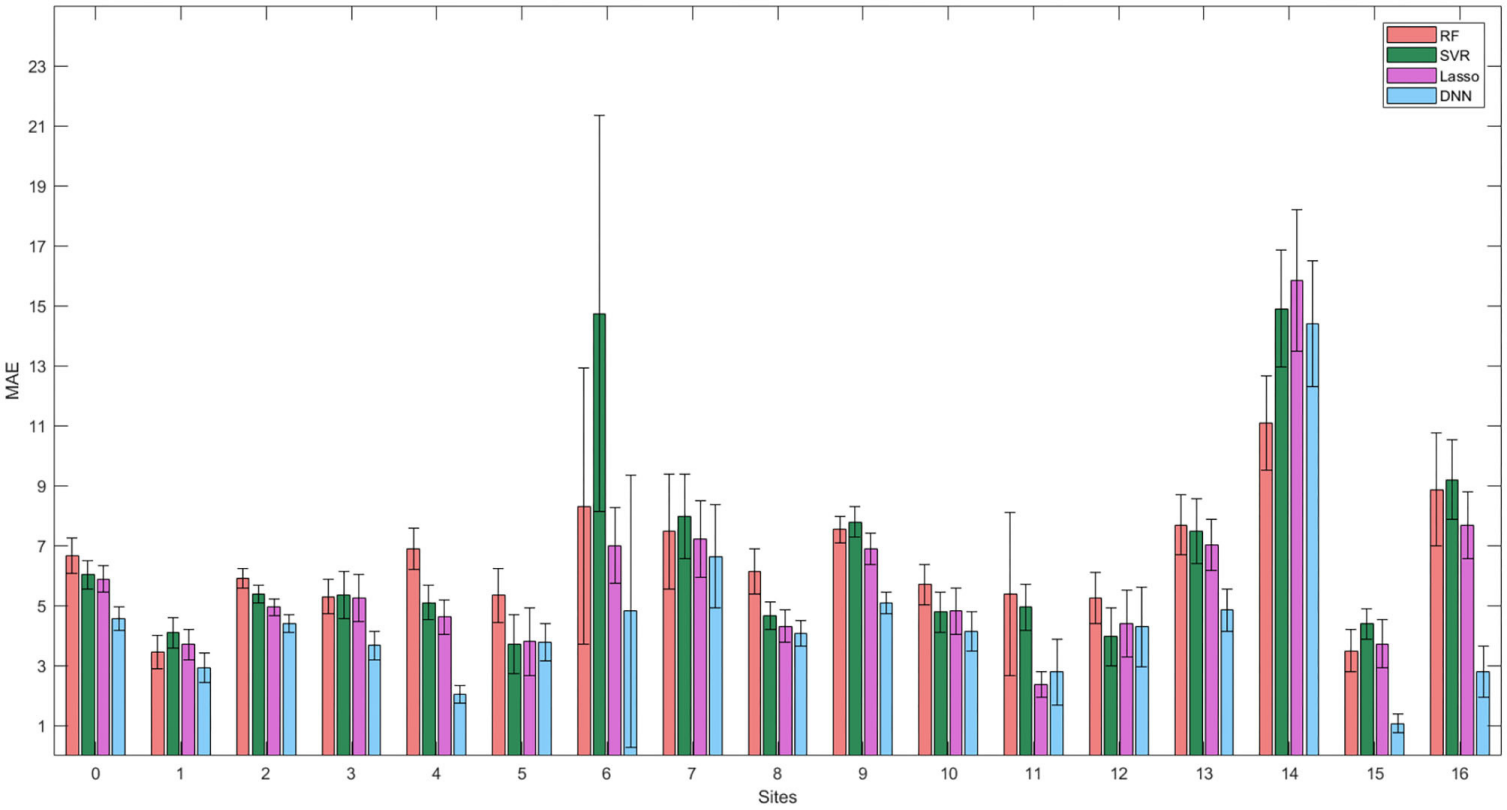
Mean values and standard errors of MAE resulting from the independent hold out test set grouped by the different sites for the four models.

It is worth noting that several samples belonging to specific sites reported systematic age underestimation or overestimation showing larger deviations from the ideal age model for all the ML regression algorithms. We better investigated the effect of the site heterogeneity on the prediction accuracy by grouping the MAE values for each site. As shown in Figure 6, the DNN models exhibit the greatest homogeneity across the sites with the exception of the site 14, which appears to be an outlier site for all the models.

In addition, we evaluated the ensemble variability as proposed in (40). This metric is assessed as the standard deviation of the prediction error within the ensemble and is related to the uncertainty in neural networks (41). We divided the 15–90 age range into 15 bins of 5 years each in order to compare uncertainty to available training sample and prediction error in different age ranges.

**Figure 7:**
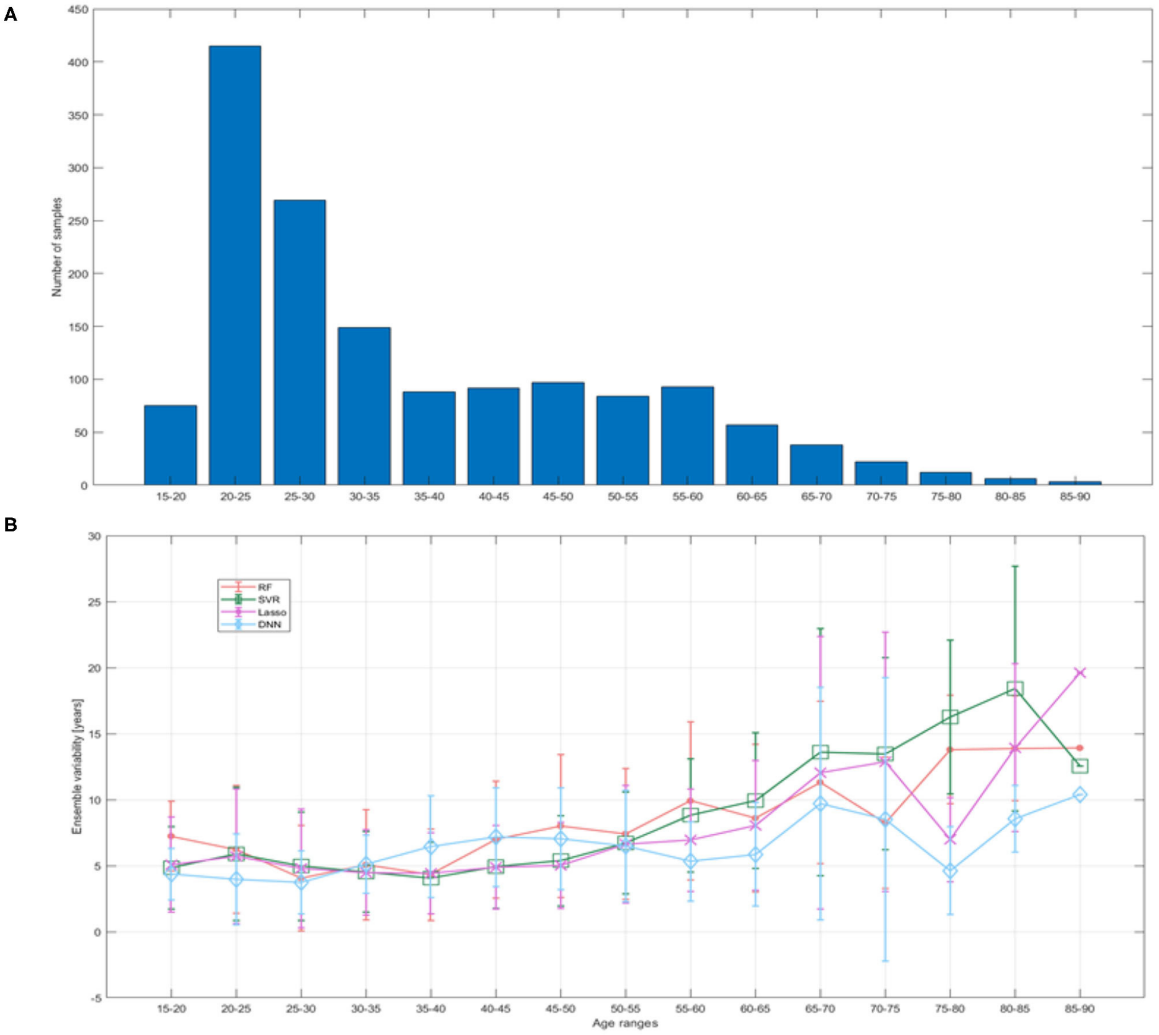
**(A)** Training sample size reported for bins of 5 years; **(B)** ensemble variability within the test set quantified as the standard deviation of the prediction error within the ensemble of models for each ML algorithm.

Figure 7 reports the mean ensemble variability as a function of age range. It is clearly evident that as the training sample decreased, the uncertainty increased and vice versa, but the DNN models show lower variability and greater stability over the age ranges with few training examples compared to other models.

### 4.3. Identification of Best Features

**Figure 8:**
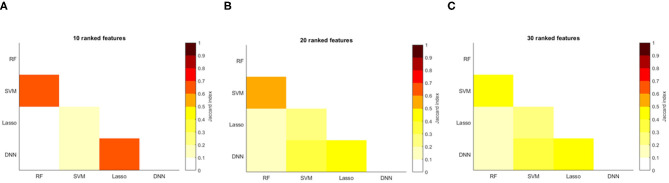
Overlapping between the top **(A)** 10 features; **(B)** 20 features; **(C)** 30 features of each couple of models.

We computed the feature ranking list resulting from each ML algorithms by applying the RRA method. The overlap between each couple of ranked list was assessed to verify the consensus between each couple of ML algorithms and for the clinical interpretability of the results. Figure 8 shows the overlapping between the feature ranking of each couple of algorithms for increasing number of features. It can be noted that for the first 10 features, DNN and Lasso show an overlap around 70%, as well as between RF and SVM. The overlap index decreases by increasing the number of features, showing how the different classification approaches actually identify different descriptors significantly associated with the age prediction. The list the most important features for the DNN models with the ranking position is reported in Table 3.

**Table 3:** Top 30 ranked features for DNN models grouped by category (R, Right; L, Left; curv, mean curvature; thick, thickness; vol, volume).

**Sub-cortical volume**	**Cortical features**	**WM volumes**	**Global features**
(5) L Thalamus	(1) L superiorfrontal thick	(10) wm L transversetemporal	(9) WM hypointensities
(6) R Thalamus	(11) L superiorfrontal vol	(18) wm R precentral	(3) Brain Stem
(13) L Putamen	(2) R superiorfrontal thick		(27) SubCortGrayVol
(16) R Putamen	(12) R superiorfrontal vol		
(7) 3rd Ventricle	(8) L lateraloccipital thick		
(14) L Lateral Ventricle	(17) R rostralmiddlefrontal thick		
(15) R Lateral Ventricle	(19) L inferiortemporal curv		
(22) L choroid plexus	(20) R inferiortemporal curv		
(23) R choroid plexus	(4) L transversetemporal curv		
	(21) R caudalanteriorcingulate curv		
	(26) R posteriorcingulate curv		
	(28) R cuneus curv		
	(24) R superiorfrontal vol		
	(25) R parsorbitalis vol		
	(29) L superiorparietal curv		
	(30) L lateralorbitofrontal curv		

*The ranking position for each feature is reported in brackets*.

## 5. Discussion

In this work we applied different ML algorithms to predict the brain age of 2,170 healthy subjects by using the morphological features extracted from T1-weighted MRI provided during the Predictive Analytic Competition 2019. Our results highlight that the DNN models achieved the best performance with *MAE* = 4.6 on the hold-out test, outperforming the other regression strategies.

The prediction accuracy we obtained compares favorably with other studies in which several morphological measures have been used to predict brain age (0.6 < *R* < 0.9 and 4 < *MAE* < 6) (3, 5, 42–47). Most of these studies are focused on younger subjects (age < 20 years) and reported MAE < 2 (42, 43, 46, 47), while other works showed that the prediction error increases with increasing age with MAE> 3 (6, 44, 45). For example, (3) obtained a *MAE* = 4 year by using a sample with subjects aged from 45 to 91, while (44) reported lower accuracy for the older group with MAE ranging between 1.57 (for the 8−18 age range) and 5.5 (for the oldest group in 65–96 age range) with neural networks using all the morphological descriptors. In our very recent works we obtained *MAE* = 2.2 with complex network modeling (7) and *MAE* = 2.5 with morphological features (21) on ABIDE dataset (6–40 years).

Several solutions have been proposed to overcome these limitations. As an example, (48) proposed a completely automated pipeline that can find the most appropriate model for the dataset under analysis and provide a complete comparison with the most commonly used models. Different models and their hyperparameters are extensively tested to provide the optimal model for the training dataset.

Other much more complex models in conjunction with different techniques have been proposed with the aim of generalizing the predictive models and making them as independent as possible from the training database. Peng et al. (49) developed a Simple Fully Convolutional Network (SFCN) architecture that uses 3D minimally-preprocessed T1 brain image for brain age prediction. Their model achieved state-of-the-art *MAE* = 2.14 years in the UK Biobank dataset (14,503 subjects, of which 12,949 are used for training) by using proper data augmentation and regularization techniques. They also used their trained models on the dataset provided by Predictive Analysis Competition 2019 resulting the best team with *MAE* = 2.90 years. (40) proposed an ensemble of CNN models trained and tested on an minimally processed T1 MRI scans of 10,176 subjects collected from 15 large-scale open-access databases in order to produce a result that is more robust to scanner's type, field strength, and resolution. The authors showed that by using both CNN models and data augmentation the results improved with *MAE* = 3.07 years and a correlation between chronological and predicted age of *R* = 0.98. These architectures employ raw high-dimensional data and have been proven to be particularly effective in learning relevant representations and latent relationships among raw data and outcomes. Indeed, convolutional neural networks can perform predictions directly from unprocessed neuroimaging data, thus overcoming some image processing steps, reducing pre-processing time and eliminating the feature engineering phase (8). On the other hand, here we exploited a feature-based learning approach based on morphological features extracted by using the FreeSurfer software. FreeSurfer has been widely adopted by scientific communities to investigate the effects of several disorders on morphological age-related brain changes (5, 50, 51), hence having both neurodevelopmental and aging models based on such features could improve the identification of normal trajectories, which could be used in turn, for example, to compare different studies and several diseases and to assess more accurately potential morphological abnormalities linked to a specific condition.

A salient point is the model homogeneity with respect to the demographic characteristics of the samples such as age range and acquisition sites. Indeed, reporting a constant behavior across acquisition sites and for different age bins is important to ensure the reliability and generalization of the ML models. The second aim of the PAC 2019 Challenge was to minimize the Spearman correlation coefficient between the age gap and the chronological age in order to achieve an unbiased algorithm for brain age prediction. Figures 4, 5 show that although the DNN models exhibit the lowest correlation values (*R* = 0.38 for cross-validation and *R* = 0.4 for the independent test), a systematic age underestimation in the age range 60−90 and overestimation in the age range 20−35 can be noted. This finding indicates that age bias correction techniques need to be further applied to achieve less biased models (52, 53).

Regarding the homogeneity behavior of the learning algorithm across sites, some methods have been proposed to minimize the effect of the sites. In the work of (54), this aspect has been tackled specifically through the strategy of transfer learning: the authors trained CNN models on a dataset of healthy Icelanders and tested on the two datasets IXI and UK Biobank, reporting *MAE* = 3.39 and *R*^2^ = 0.87. These works highlight that significant improvements can also be achieved by greatly expanding the sample size and by using approaches such as ensemble prediction models.

Here we tested the performance heterogeneity across sites and prediction uncertainty. Model uncertainty can be seen as the lack of confidence in the prediction caused by the model's failure to catch the true data generation process (41). Here, uncertainty was measured by calculating the prediction variability within the ensemble. Figure 7 shows that the DNN models exhibit lower variability where training sample decreased in contrast to the other ML strategies. Moreover, our results point out that the proposed DNN architecture shows lower MAE consistently across all sites, except for one site that was found to be an outlier for all machine learning algorithms (see Figure 6). We applied a robust consensus strategy to identify the final ranked features for each algorithm. Our analysis had the two-fold purpose of providing a clinical interpretability for the most performing models and explaining the different performance of the algorithms through the comparison of the most important predictors for each strategy. Figure 8 clearly shows that the ranked list of the most important features for the DNN model is different from the other strategies, except for a higher overlap of the first 10 most important features with those of the Lasso algorithm. Such overlap could also explain the performance of Lasso method which resulted the second best performing algorithm with *MAE* = 5.8.

Table 3 shows the most relevant features for age prediction: we found morphological attributes of superior frontal, middle frontal and cingulate cortical regions among the most important features. Our findings are consistent with previous works in which brain changes have been related to age in frontal lobe, several parietal regions, cingulate cortex, brainstem and sub-gyral regions (46, 47, 55–59) Moreover, both putamen and thalamus volumes have been identified as important predictors. In the literature, the impact of age on different subcortical brain volumes have been thoroughly studied, revealing heterogeneous age responses for thalamus, caudate, hippocampus and cerebellar white and gray matter (60–62). In particular, the integrity and size of the thalamic nuclei were found to correlate negatively with age and with the ability to perform attention and memory cognitive processes (63). Interestingly, for our DNN models the ventricles and choroid plexus were identified among the most relevant for age prediction. These findings are particularly in agreement with studies describing the brain's fluid-filled ventricles as a biomarker of the aging brain (64, 65). We found an high overlap with the regions identified in the work of (40). The authors used CNN models in conjunction with explainable AI techniques to derive explanation map which highlighted a major contribution of ventricles and cisterns. Here we also identified the choroid plexus that represents the principal source of cerebrospinal fluid (CSF), whose expansion have been associated to decrease in WM/GM volumes, resulting a reliable aging marker and index for brain atrophy (66).

It is worth noting that although the automated segmentation techniques such as those provided by FreeSurfer software have been proved effective in detecting longitudinal changes and have been used for studying brain development and aging (67), here we found lower performance compared to other strategies adopting convolutional neural networks such as the algorithm proposed by the winner of the challenge. Indeed, the FreeSurfer automated segmentation methods exploit probabilistic atlas generated from a set of manually labeled T1-weighted scans that return information about the shape and location of the brain areas. Hence the segmentation accuracy may depend on several factors such as age (68) and brain size (69), highlighting the need to reduce bias and improve accuracy of automated segmentation models. These limitations are overcame by the winning model that leverages single voxel-based information. It is interesting to note that the authors achieved better performance by adding white matter and gray matter maps to the raw scans, proving that the information contained in the two maps would be complementary to that provided by the raw scans and useful to refine the proposed predictive model.

## 6. Conclusion

In this work we tested the effectiveness of a DNN architecture to predict the brain age by using the morphological features extracted from the T1-weighted images of 2,170 subjects during the Predictive Analytic Competition 2019. We extensively evaluated different aspects of the proposed architecture by comparing both performance with other commonly used ML algorithms and by proposing a robust rank aggregation scheme to derive the most important features. Besides the best performing algorithm, the DNN model we proposed shows important differences with the other ML algorithms: the lower ensemble variability suggests that the DNN architecture can be consistently used to estimate age even when datasets exhibit non-homogeneous age distribution over the age range. Moreover, the low-overlap with the most important features selected by the other methods indicates that the DNN models could provide different indications on the morphological aging mechanisms by identifying reliable imaging biomarkers. In our work we presented a comparison of a DNN architecture with other more widespread regression algorithms, however other approaches such as XGBoost models could be investigated for further analysis. Furthermore, here we performed a partial tuning of the DNN parameters, while a refinement of the tuning procedure could improve the accuracy of the models. The proposed models could be further improved by applying age bias correction methods and by using an higher number of samples to ensure the generalization of results.

## Data Availability Statement

Publicly available datasets were analyzed in this study. This data can be found here: https://web.archive.org/web/20200214101600/https://www.photon-ai.com/pac2019.

## Author Contributions

AL and ST conceived the analysis. AL performed the data curation, implemented the software pipelines, and performed the analysis. AL, ST, and RB defined the methodology. GD set the computational resources. RB and ST supervised the analysis. AL, ST, RB, NA, and AM analyzed and interpreted the results. AL and ST wrote the original draft. AL, ST, RB, NA, AM, and GD edited the final version of the paper. All authors have approved the final version of the manuscript.

## Conflict of Interest

The authors declare that the research was conducted in the absence of any commercial or financial relationships that could be construed as a potential conflict of interest.

## Footnotes

^1^https://web.archive.org/web/20200214101600/; https://www.photon-ai.com/pac2019

^2^https://www.recas-bari.it/index.php/en/

^3^http://www.pon-recas.it

^4^https://surfer.nmr.mgh.harvard.edu/

^5^https://surfer.nmr.mgh.harvard.edu/fswiki/recon-all

^6^https://cran.r-project.org/web/packages/caret/caret.pdf

^7^https://cran.r-project.org/web/packages/randomForest/randomForest.pdf

^8^https://cran.r-project.org/web/packages/h2o/h2o.pdf
